# Synergistic anticancer effects of triptolide and celastrol, two main compounds from thunder god vine

**DOI:** 10.18632/oncotarget.5411

**Published:** 2015-10-05

**Authors:** Qi-Wei Jiang, Ke-Jun Cheng, Xiao-Long Mei, Jian-Ge Qiu, Wen-Ji Zhang, You-Qiu Xue, Wu-Ming Qin, Yang Yang, Di-Wei Zheng, Yao Chen, Meng-Ning Wei, Xu Zhang, Min Lv, Mei-Wan Chen, Xing Wei, Zhi Shi

**Affiliations:** ^1^ Department of Cell Biology & Institute of Biomedicine, National Engineering Research Center of Genetic Medicine, Guangdong Provincial Key Laboratory of Bioengineering Medicine, College of Life Science and Technology, Jinan University, Guangzhou, Guangdong, China; ^2^ Chemical Biology Center, Lishui Institute of Agricultural Sciences, Lishui, Zhejiang, China; ^3^ National First-Class Key Discipline for Traditional Chinese Medicine of Nanjing University of Chinese Medicine, Nanjing, Jiangsu, China; ^4^ Institute of Materia Medica, Zhejiang Chinese Medical University, Hangzhou, Zhejiang, China; ^5^ State Key Laboratory of Quality Research in Chinese Medicine, Institute of Chinese Medical Sciences, University of Macau, Macau, China

**Keywords:** triptolide, celastrol, combination therapy, cancer

## Abstract

Triptolide and celastrol are two main active compounds isolated from Thunder God Vine with the potent anticancer activity. However, the anticancer effect of triptolide in combination with celastrol is still unknown. In the present study, we demonstrated that the combination of triptolide with celastrol synergistically induced cell growth inhibition, cell cycle arrest at G2/M phase and apoptosis with the increased intracellular ROS accumulation in cancer cells. Pretreatment with ROS scavenger N-acetyl-L-cysteine dramatically blocked the apoptosis induced by co-treatment with triptolide and celastrol. Treatment with celastrol alone led to the decreased expressions of HSP90 client proteins including survivin, AKT, EGFR, which was enhanced by the addition of triptolide. Additionally, the celastrol-induced expression of HSP70 and HSP27 was abrogated by triptolide. In the nude mice with xenograft tumors, the lower-dose combination of triptolide with celastrol significantly inhibited the growth of tumors without obvious toxicity. Overall, triptolide in combination with celastrol showed outstanding synergistic anticancer effect *in vitro* and *in vivo*, suggesting that this beneficial combination may offer a promising treatment option for cancer patients.

## INTRODUCTION

**Figure 1:**
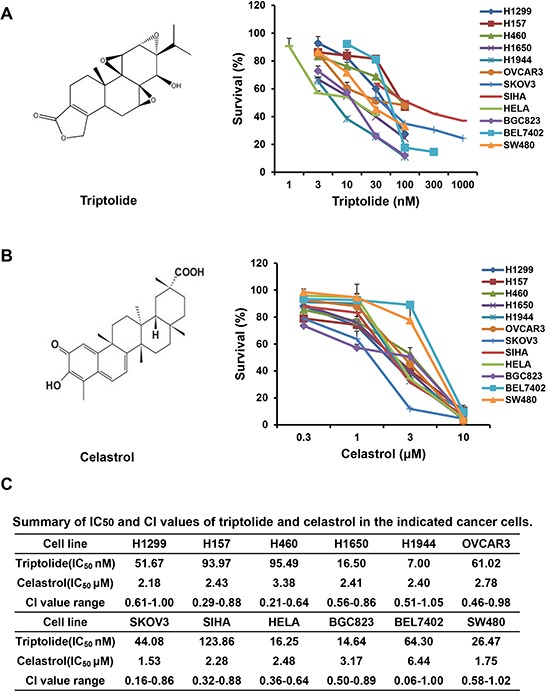
Alone or co-treatment with triptolide and celastrol inhibit the growth of cancer cells *in vitro* Cells were treated with the indicated concentrations of triptolide or celastrol for 72 h, and cell survival was determined by MTT assay. The chemical structure of triptolide **A.** celastrol **B.** and summary survival curves, IC_50_ and CI values in the indicated cancer cells **C.** were shown.

Triptolide (Figure 1A) and celastrol (also known as tripterine, Figure 1B) are two main compounds from Thunder God Vine (also known as *Tripterygium wilfordii* and Lei Gong Teng) with a broad range of bioactivities, especially anticancer activity [1]. Numerous studies have reported that both triptolide and celastrol have the anticancer effects by inducing cell cycle arrest and apoptosis in various cancer cells *in vitro* and *in vivo* [2–14]. Mechanistically, triptolide directly binds to the subunit of transcription factor 2 (TFIIH), excision repair cross-complementation group 3 (ERCC3, also known as XPB), and inhibits its DNA-dependent ATPase activity, which leads to the inhibition of RNA polymerase II–mediated transcription and likely nucleotide excision repair [15]. Celastrol has been identified as a novel inhibitor of HSP90 and displays anticancer activity by inducing the degradation of HSP90 client proteins, such as AKT, EGFR, CDKs, IAPs and p53, etc [16, 17]. The identification of XPB and HSP90 as the target of triptolide and celastrol respectively accounts for the majority of their known biological activities.

Combination therapy is the routine strategy of cancer chemotherapy with significant advantages including lower treatment failure rate and slower development of drug resistance, [18]. It has reported that triptolide could sensitize cancer cell lines to several chemotherapeutic drugs *in vivo* and *in vitro*, including cisplatin, adriamycin, temozolomide and sorafenib, etc [2, 4, 6, 19]. Celastrol in combination with chemotherapeutic agents also show the synergistic anticancer effect on suppressing the proliferation of multiple type of cancers such as melanoma, hepatocellular carcinoma, breast cancer and lung cancer. [20–23]. Although triptolide and celastrol coexist in Thunder God Vine and have the different anticancer molecular mechanisms, the anticancer effect of triptolide in combination with celastrol is still unknown. In this study, we demonstrated that the combination of triptolide with celastrol had the synergistic anticancer effect *in vitro* and *in vivo*, which might be due to their complementary anticancer molecular mechanisms.

## RESULTS

### Triptolide and celastrol synergistically inhibit the growth of cancer cells *in vitro*

**Figure 2:**
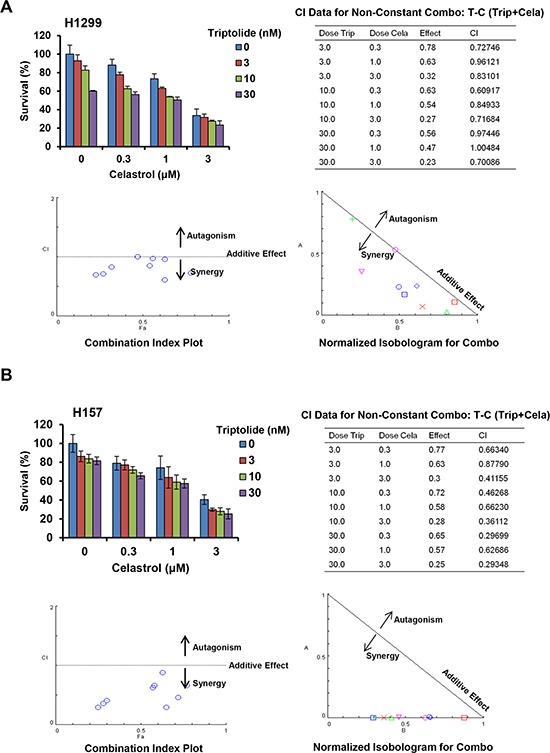
Triptolide and celastrol synergistically inhibit the growth of H1299 and H157 cancer cells *in vitro* H1299 **A.** and H157 **B.** cells were treated with the indicated concentrations of triptolide and celastrolfor 72 h, and cell survival was detected by MTT assay. The data were analyzed by CompuSyn software, and the summary growth histogram, dose-effect curve, CI values and normalized isobologram were shown. The shapes/colors respectively represent different combinations. Combination index analysis showed that a combination index of 1 reflects additive effects, whereas values greater than and less than 1 indicate antagonism and synergy, respectively. In isobologram analysis, the diagonal line represents the isoeffect line of additive. Points above this line indicate antagonism, and points below this line indicate synergy.

To assess the synergistic effect of triptolide and celastrol on cancer cells, cell survival was detected by MTT assay. As shown in Figure 1, the survival of all used cancer cells was decreased in a dose-dependent manner *in vitro* after either triptolide or celastrol treatment. The IC_50_ values of triptolide and celastrol in these cells were range from 7.00 to 123.86 nM and 1.53 to 6.44 μM, respectively. However, in human normal embryonic kidney HEK293T cells triptolide slightly inhibited cells growth with the IC_50_ > 1000 nM which is significantly higher than those of cancer cells, and celastrol considerably inhibited cells growth with the IC_50_ 2.99 μM which are equal to those of cancer cells (Supplementary Figure S1A). After co-treatment with triptolide (3, 10 and 30 nM) and celastrol (0.3, 1 and 3 μM), the survival of cancer cells were significantly reduced in comparsion with triptolide or celastrol alone treatment. Almost all CI values of combination in cancer cells were <1, suggesting that the antigrowth effect of triptolide in combination with celastrol in the indicated cancer cells is synergistic rather than additive (Figure 1C, 2 and Supplementary Figure S2). Nevertheless, only small part of CI values of combination in HEK293T cells were <1, suggesting that the synergistic effect of triptolide in combination with celastrol in normal cells is not significant as that in cancer cells (Supplementary Figure S1B).

### Triptolide and celastrol synergistically induce G2/M cell cycle arrest in cancer cells

**Figure 3:**
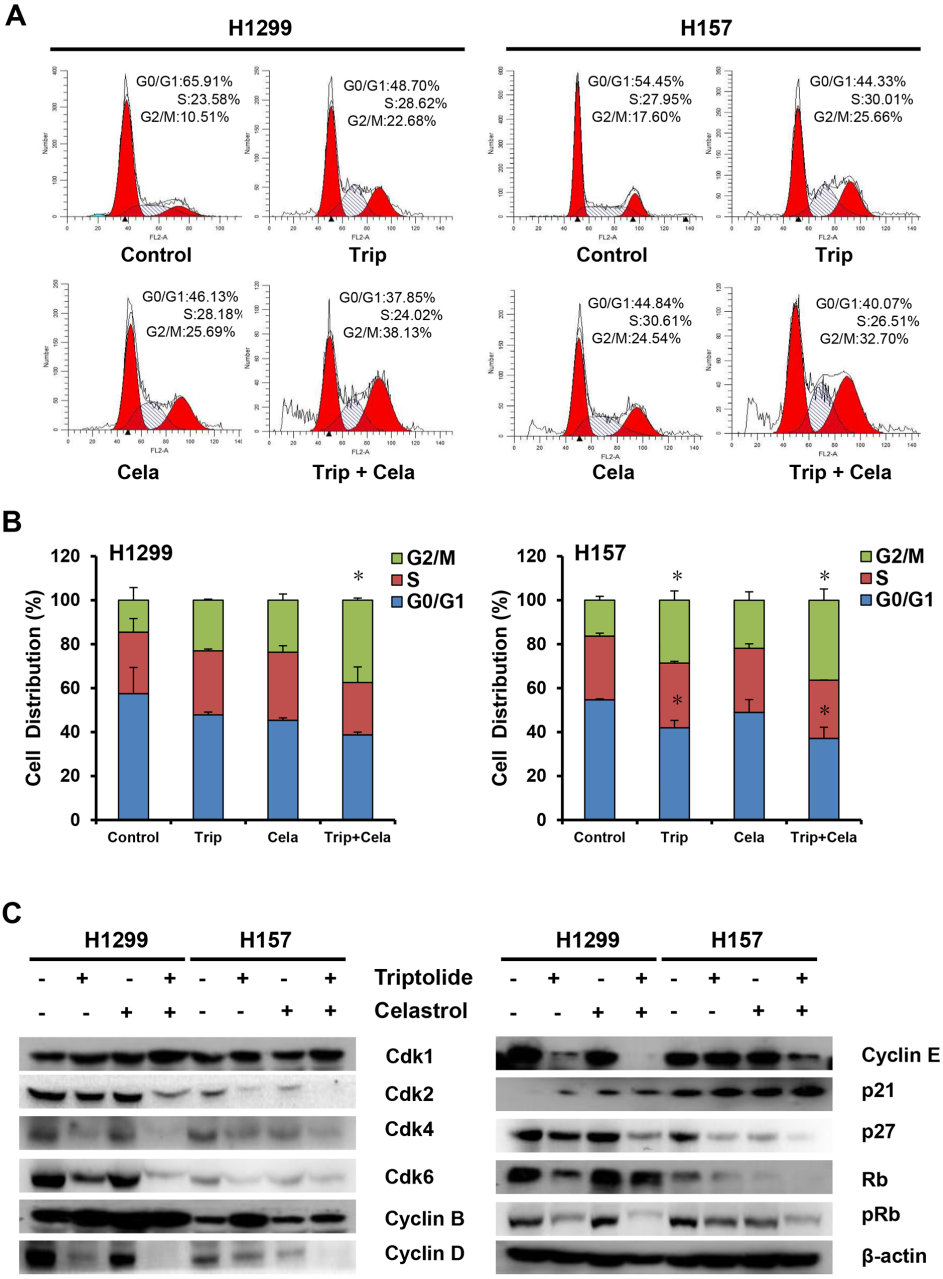
Triptolide and celastrol synergistically induce G2/M cell cycle arrest in H1299 and H157 cancer cells H1299 cells was exposed to triptolide (30 nM), celastrol (0.6 μM) or a combination of both (triptolide 30 nM + celastrol 0.6 μM), and H157 cells was exposed to triptolide (15 nM), celastrol (1.6 μM) or a combination of both (triptolide 15 nM + celastrol 1.6 μM) for 48 h. The distribution of cell cycle was detected by FCM with PI staining. The percentages of subG1, G1/G0, S, G2/M phase were calculated using ModFit LT 3.0 software. The protein expression was examined by Western blot after lysing cells, and β-actin was used as loading control. The representative charts **A.** quantified results **B.** and Western blot results **C.** of three independent experiments were shown. *P < 0.05 *vs*. corresponding control.

To determine whether the growth inhibition of cancer cells by triptolide in combination with celastrol is due to cell cycle arrest, cell cycle distribution was assessed by FCM with PI staining. As shown in Figure 3A and 3B, either triptolide or celastrol alone treatment induced the accumulation in G2/M phase and reduction in G0/G1 phase of cell population in both H1299 and H157 cancer cell lines, and co-treatment with triptolide and celastrol induced the more significant accumulation in G2/M phase and reduction in G0/G1 phase of cell population in both cancer cell lines. To investigate the molecular mechanism of cell cycle arrest by triptolide and celastrol, the cell cycle related proteins were detected by Western blot. The results showed that the protein levels of Cdk1, Cyclin B and p21, which control the transition of G2/M phase, were increased mildly in triptolide or celastrol alone group and dramatically in the combination group of triptolide with celastrol. Additionally, the protein levels of Cdk2/4/6, Cyclin D/E, pRb, Rb and p27 were decreased moderately in either triptolide or celastrol alone group and significantly in the combination group of triptolide with celastrol (Figure 3C and Supplementary Table S1). Taken together, our data indicate that triptolide and celastrol can synergistically induce G2/M cell cycle arrest in cancer cells.

### Triptolide and celastrol synergistically induce cancer cell apoptosis

**Figure 4:**
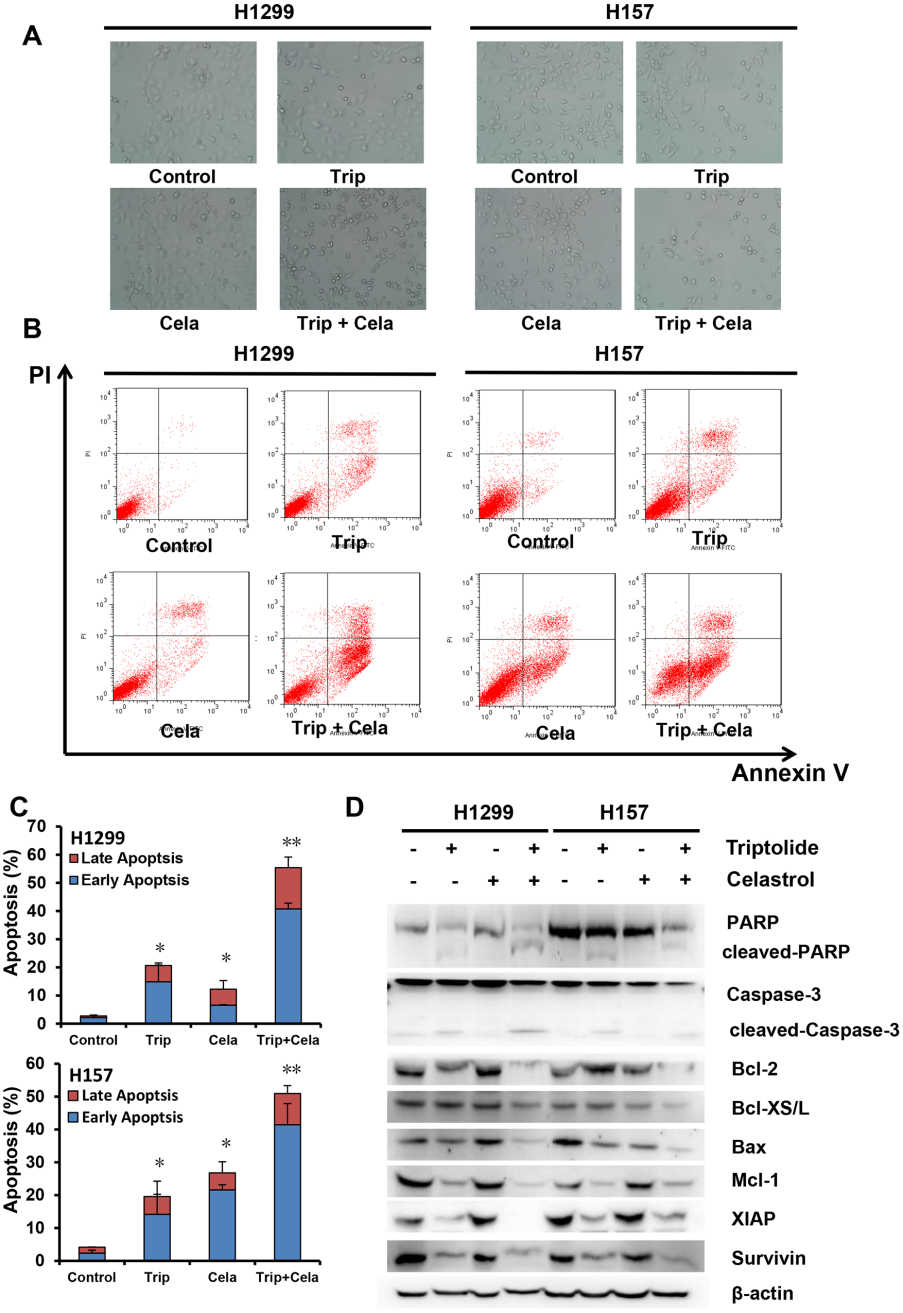
Triptolide and celastrol synergistically induce apoptosis in H1299 and H157 cancer cells H1299 and H157 cells were treated similarly as described in Figure 3 for 48 h, and photographed under microscope. The representative micrographs **A.** of the cellular morphological signatures after treatments were shown. The apoptosis was detected by FCM Annexin V/PI staining. The proportions of Annexin V+/PI− and Annexin V+/PI+ cells indicated the early and late stage of apoptosis. The protein expression was examined by Western blot after lysing cells, and β-actin was used as loading control. The representative charts **B.** quantified results **C.** and Western blot results **D.** of three independent experiments were shown. *P < 0.05 and **P < 0.01 *vs*. corresponding control.

In addition to the evaluation of triptolide in combination with celastrol induced growth inhibition and cell cycle arrest, their effects on apoptosis were analyzed by microscopy and FCM. As shown in Figure 4A, either triptolide or celastrol alone treatment conduced a few cells undergoing apoptosis with a characteristic morphology such as cell shrinkage and rounding in both H1299 and H157 cancer cell lines, and co-treatment with triptolide and celastrol conduced more apoptosis in both cancer cell lines. The apoptosis was further quantified by FCM with Annexin V/PI staining. The results showed triptolide or celastrol alone treatment caused moderate apoptosis including early (Annexin V positive and PI negative) and late (Annexin V positive and PI postive) apoptosis in both H1299 and H157 cancer cell lines, and co-treatment with triptolide and celastrol caused significant total apoptosis in both cancer cell lines (Figure 4B and 4C). To investigate the molecular mechanism of cell apoptosis by triptolide and celastrol, the apoptotic related proteins were detected by Western blot. As shown in Figure 4D and Supplementary Table S1, the markers of apoptosis, the cleaved PARP and Caspase-3, were clearly generated in the co-treatment group compared with either single drug group. Furthermore, the combination group of triptolide with celastrol showed the obvious decreased expression levels of pro-apoptotic proteins including Bax and Bcl-XS/L and anti-apoptotic proteins including Bcl-2, Mcl-1, survivin and XIAP in comparison with triptolide or celastrol alone group. In conclusion, these results suggested that triptolide and celastrol can synergistically induce cancer cells apoptosis.

### ROS is critical for the synergistic anticancer effects of triptolide and celastrol

**Figure 5:**
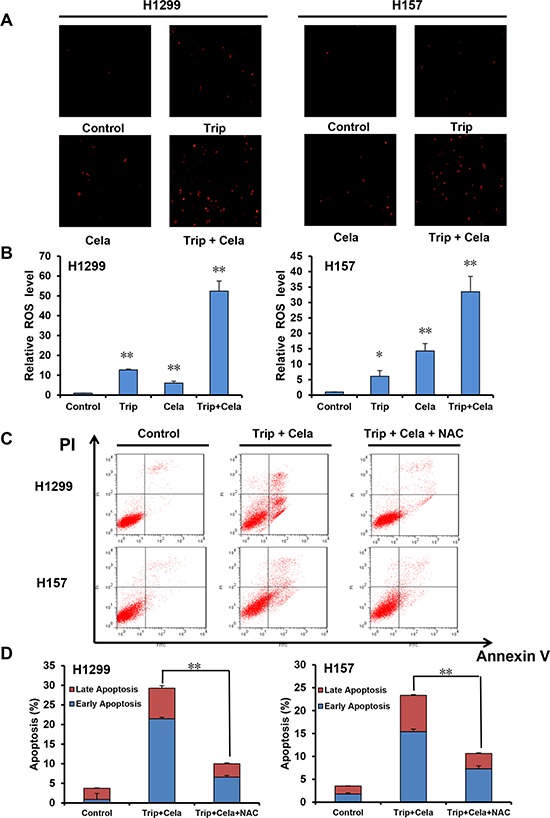
ROS is critical for the synergistic anticancer effects of triptolide and celastrol H1299 and H157 cells were treated similarly as described in Figure 3 for 24 h, stained with DHE and photographed under fluorescent microscope. The representative micrographs **A.** and quantified results **B.** of three independent experiments were shown. Cells were co-treated with triptolide and celastrol for 24 h in the present or absent of 3 mM NAC pretreatment for 1 h. The apoptosis was detected by FCM with Annexin V/PI staining. The proportions of Annexin V+/PI− and Annexin V+/PI+ cells indicated the early and late stage of apoptosis. The representative charts **C.** and quantified results **D.** of three independent experiments were shown. *P < 0.05 and **P < 0.01 *vs*. corresponding control.

ROS plays a critical role in mediating numerous anticancer agents executing anticancer effects [24]. We next assess the role of ROS in the synergistic anticancer effects of triptolide and celastrol. Dihydroethidium (DHE) is a classic ROS fluorescent probe, which can penetrate through living cell membrane freely and be oxidized by intracellular ROS to oxide ethidium that conjugated with DNA to emit the detectable red fluorescence [25]. As shown in Figure 5A and 5B, compared with triptolide or celastrol alone treatment, co-treatment with triptolide and celastrol prominently led to the enhancement of DHE fluorescent intensities in both H1299 and H157 cancer cells, suggesting the intracellular ROS levels of co-treatment group were significantly higher than those of either single drug group in both cancer cells. Previous studies have showed that ROS levels positively correlate with cell apoptosis, and the antioxidative agent NAC can prevent the production of ROS to suppress cell apoptosis [25]. To further investigate the relationship between the ROS generation and apoptosis induced by the combination of triptolide with celastrol, cells were co-treated with triptolide and celastrol for 24 h in the presence or absence of 3 mM NAC pretreatment for 1 h. The cell apoptosis was detected by FCM with Annexin V/PI staining, and the results demonstrated that cell apoptosis of the combination therapy was mostly blocked by NAC in both H1299 and H157 cancer cells (Figure 5C and 5D). In brief, these data indicate that ROS is critical for the synergistic anticancer effects of triptolide and celastrol.

### Combinational effect of triptolide and celastrol on heat shock proteins and their client proteins

**Figure 6:**
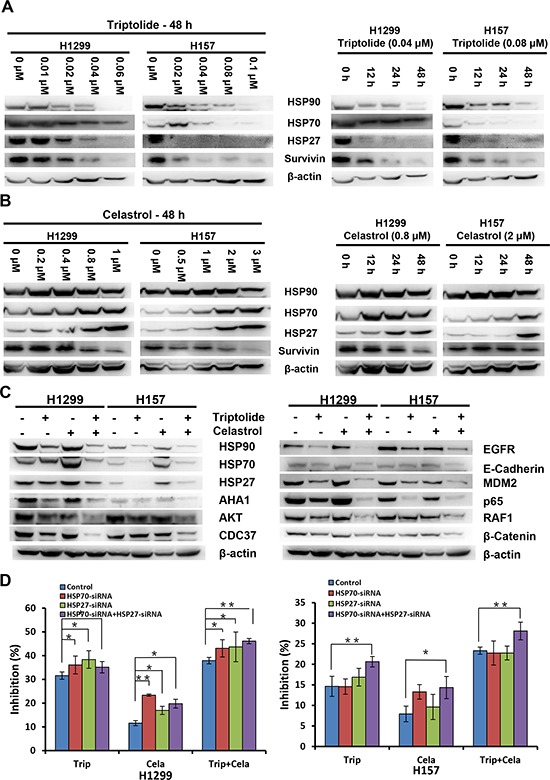
Combinational effect of triptolide and celastrol on heat shock proteins and their client proteins H1299 and H157 cells were treated with the indicated concentrations and time-points of triptolide **A.** and celastrol **B.** alone, or similarly as described in Figure 3 for 48 h **C.** Knockdown of HSP70 and HSP27 enhances the antigrowth effects of triptolide in combination with celastrol **D.** The protein expression was examined by Western blot after lysing cells, and β-actin was used as loading control. The representative Western blot results of three independent experiments were shown. *P < 0.05 and **P < 0.01 *vs*. corresponding control.

To further elucidate the molecular mechanism of the synergistic anticancer effects of triptolide and celastrol, we focused on the molecular targets of triptolide and celastrol, especially heat shock proteins (HSPs) and their client proteins by detecting their protein levels with Western blot. As shown in Figure 6A, triptolide alone treatment dose- and time-dependently reduced the protein levels of HSP27, HSP70, HSP90 and its client protein survivin in both H1299 and H157 cancer cells. However, celastrol alone treatment in a dose- and time-dependent manner decreased the protein levels of survivin, but increased the protein levels of HSP27 and HSP70 in both cancer cells (Figure 6B). Moreover, the addition of triptolide prevented the increase of HSP27 and HSP70 proteins by celastrol, and synergized with celastrol to decrease the protein levels of HSP90, co-chaperone proteins CDC37 and AHA1, client proteins survivin, EGFR, RAF1, AKT, MDM2, β-catenin, and other proteins E-Cadherin and p65 (Figure 6C) and Supplementary Table S1. To investigate whether knockdown of HSP70 and HSP27 by siRNA affects the synergistic anticancer effect of triptolide in combination with celastrol, we silenced the expression of HSP70 and HSP27 alone or combination by transfection of their specific siRNA into both H1299 and H157 cells. The results showed that in H1299 cells knockdown of HSP70 and HSP27 alone or combination by siRNA significantly enhanced the growth inhibitory effects of triptolide and celastrol alone or combination, and in H157 cells only knockdown of both HSP70 and HSP27 by siRNA significantly enhanced the growth inhibitory effects of triptolide and celastrol alone or combination (Figure 6D).

### Triptolide and celastrol synergistically inhibit xenograft tumor growth in nude mice

**Figure 7:**
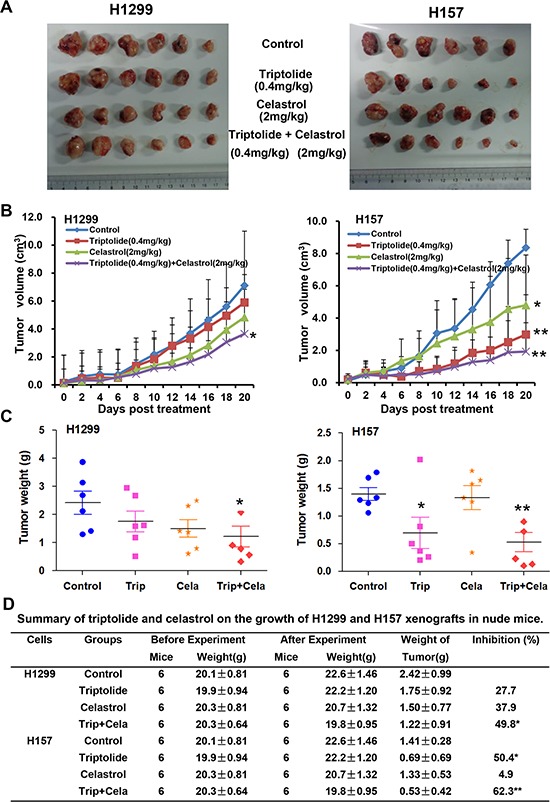
Triptolide and celastrol synergistically inhibit xenograft tumor growth in nude mice Each mouse was injected subcutaneously with H1299 or H157 cells (3 × 10^6^ in 100 μL of medium) under the shoulder. When the subcutaneous tumors were approximately 0.5 × 0.5 cm^2^ (two perpendicular diameters) in size, mice were randomized into four groups, and were injected intraperitoneally with vehicle alone (saline with 0.1% DMSO), triptolide alone (0.4 mg/kg), celastrol alone (2 mg/kg), or a combination of triptolide and celastrol every two day. The body weights of mice and tumor volume were recorded. The mice were anaesthetized after experiment, and tumor tissue was excised from the mice and weighted. The original tumors **A.** tumor volumes **B.** tumor weights **C.** and summary data **D.** were shown. The values presented are the means ± SD for each group. *P < 0.05 and **P < 0.01 *vs*. corresponding control.

To examine the synergistic antitumor effects of triptolide and celastrol *in vivo*, we generated the xenograft tumor models by transplanting H1299 and H157 cancer cells into nude mice. As shown in Figure 7A–7C, compared with triptolide or celastrol alone treatment, co-treatment with triptolide and celastrol significantly inhibited the growth of both H1299 and H157 tumors by diminishing the volume and weight of tumors. The inhibition rates of tumor growth in the co-treatment group were 49.8% (H1299) and 62.3% (H157) respectively, which were obviously higher than those in either single treatment group (Figure 7D). In addition, the weight of mice did not show the statistical difference between before and after experiments in each group, suggesting neither single treatment nor co-treatment of triptolide and celastrol resulted in significant side effects in mice (Figure 7D). In short, these results indicate that triptolide and celastrol can synergistically inhibit tumor growth *in vivo*.

## DISCUSSION

Natural products play an important role in the prevention and treatment of cancer and other disease in the world [26–28]. In the present study, we demonstrated that the combination of triptolide with celastrol synergistically induced cell growth inhibition, cell cycle arrest at G2/M phase and apoptosis with the increased intracellular ROS accumulation in cancer cells. It has reported that triptolide can induce ROS generation and subsequently result in cancer cells apoptosis, and pretreatment with ROS scavenger NAC only partially suppresses triptolide-induced apoptosis, suggesting triptolide is able to induce both ROS dependent and independent apoptosis [29]. Treatment with celastrol also enhances the intracellular ROS generation to trigger apoptosis in cancer cells, while blocking of ROS accumulation with NAC completely inhibits celastrol-induced apoptosis, suggesting celastrol only induce ROS dependent apoptosis [30, 31]. In our case, co-treatment with triptolide and celastrol prominently led to the enhancement of intracellular ROS in comparisonwith either single drug treatment in cancer cells, and NAC pretreatment dramatically but not totally blocked the apoptosis induced by co-treatment with triptolide and celastrol. Our findings indicate that co-treatment with triptolide and celastrol can induce both ROS dependent and independent apoptosis, which may be due to the above different roles of ROS in triptolide- and celastrol-induced apoptosis.

**Figure 8:**
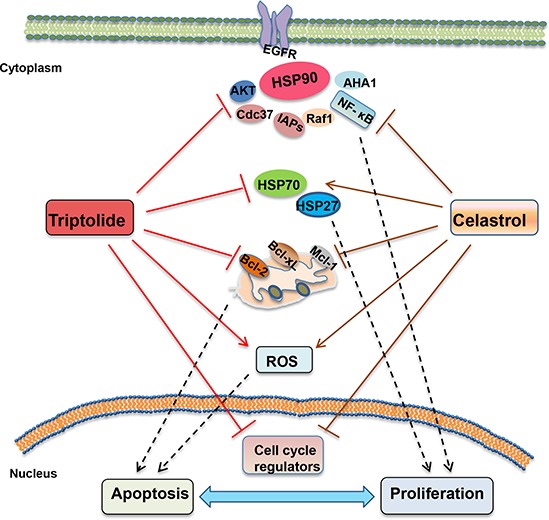
The schematic of molecular mechanisms underlying the synergistic anticancer effects of triptolide and celastrol The combination of triptolide with celastrol synergistically induced cell growth inhibition, cell cycle arrest at G2/M phase and apoptosis with the increased intracellular ROS accumulation in cancer cells. Celastrol alone led to the decreased expressions of HSP90 client proteins including survivin, AKT, EGFR, which was enhanced by the addition of triptolide. Additionally, the celastrol-induced expression of HSP70 and HSP27 was abrogated by triptolide.

Heat shock proteins (HSPs) are commonly overexpressed in a wide variety of cancers, and high levels of HSPs are closely associated with poor prognosis and treatment resistance in cancer patients as these proteins protect cancer cells against the toxic effects of genomic instability, aberrant microenviroment and therapeutic stressors including radiotherapy, thermotherapy and chemotherapy [32]. Therefore, targeting HSPs has recently emerged as a promising potential anticancer strategy. It has demonstrated that triptolide is an inhibitor of HSPs transcription which leads to enhancement of stress-induced cell death [33]. Triptolide can inhibit both mRNA and protein levels of HSP70, and induce apoptosis in HSP70 overexpressed pancreatic cancer cells [7]. Celastrol directly inhibits HSP90 activity but stressedly induce the upregulation of HSP70 [5, 34]. In our study, treatment with celastrol alone resulted in the decreased expressions of HSP90 client proteins including survivin, AKT, EGFR, etc, which was enhanced by the addition of triptolide. Additionally, the celastrol-induced upregulation of HSP70 and HSP27 was abrogated by triptolide. Furthermore, knockdown of HSP70 and HSP27 enhanced the antigrowth effects of triptolide in combination with celastrol. As two main stress-inducible HSPs, HSP70 and HSP27 are two powerful chaperones which inhibit critical effectors of the apoptotic machinery to allow cell survival under stress conditions, and overexpression of HSP70 or HSP27 in cancer cells drive cancer growth, treatment resistance and metastatic potential [35]. Therefore, downregulation of the celastrol-induced expression of HSP70 and HSP27 by triptolide may be an important mechanism of the synergistic anticancer effects of triptolide and celastrol. In addition, inhibition of HSP90 client proteins and induction of the intracellular ROS accumulation can be other reasons for the synergistic anticancer effect of triptolide and celastrol (Figure 8).

In summary, our studies provide strong evidence that combinational treatment with triptolide and celastrol at low concentration synergistically inhibit cancer cells growth by inducing cell cycle arrest and apoptosis *in vitro* and *in vivo*. Triptolide in combination with celastrol shows outstanding synergistic anticancer effect, suggesting this beneficial combination may offer a promising treatment option for cancer patients.

## MATERIALS AND METHODS

### Cells, cell culture, and reagents

Human cancer cell lines (H1299, H157, H460, H1650, H1944, OVCAR3, SKOV3, SIHA, HELA, BGC823, BEL7402, SW480) and human normal embryonic kidney cell line HEK293T were cultured in Dulbecco's modified Eagle's medium (DMEM) supplemented with 10% fetal bovine serum (FBS), penicillin (100 U/ml) and streptomycin (100 ng/ml) in a humidified incubator at 37°C with 5% CO2. Triptolide (Trip) and celastrol (Cela) from Shanghai Tauto Biotechnology were dissolved as a stock solution 10 mM in DMSO and stored at −20°C. N-acetly-L-cysteine (NAC) and dihydroethidium (DHE) were purchased from Sigma-Aldrich. Anti-AKT (4691), Anti-Bax (2772), Anti-E-Cadherin (3195), Anti-HSP27 (2402), Anti-Mcl-1 (4572), Anti-PARP (9542), Anti-Rb pS780 (9307), Anti-Surivin (2808), Anti-XIAP (2045), Anti-β-Catenin (9582) and Anti-β-Actin (4967) antibodies were from Cell Signaling Technologies. Anti-Bcl-2 (SC-492), Anti-Bcl-xS/L (SC-634), Anti-Caspase-3 (SC-1148), Anti-CDC37 (SC-13129), Anti-CDK2 (SC-163), Anti-CDK4 (SC-260), Anti-Cycin D1 (SC-718), Anti-Cyclin E (SC-481), Anti-EGFR (SC-03), Anti-HSP70 (SC-69705), Anti-HSP90 (SC-13119), Anti-MDM2 (SC-965), Anti-p65 (SC-372) and Anti-RAF1 (SC-133) antibodies were from Santa Cruz Biotechnology. Anti-p21 (610233), Anti-p27 (610241), Anti-Cyclin B (610219) and Anti-CDK1 (610037) antibodies were from BD Biosciences. Anti-CDK6 (3524-1) and Anti-Rb (2655–1) antibodies were from Abcam. Anti-AHA1 (A2617) antibody was from Abclonal. Anti-GAPDH (LK9002T) antibodies were from Tianjin Sungene Biotech.

### Cell viability assay

Cells were firstly seeded into a 96-well plate at a density of 5000 cells per well, and incubated with drugs in three parallel wells for 72 h. Then MTT was added to each well at a final concentration of 0.5 mg/ml. After incubation for 4 h, formazan crystals were dissolved in 100 μl of DMSO, and absorbance at 570 nm was measured by plate reader. The concentrations required to inhibit growth by 50% (IC_50_) were calculated from survival curves using the Bliss method. For drug combination experiments, cells were co-treated with different concentrations of triptolide and celastrol for 72 h. The data were analyzed by CompuSyn software with the results showed as combination index (CI) values according to the median-effect principle, where CI <1, =1, and >1 indicate synergism, additive effect, and antagonism, respectively. The equation for the isobologram is shown as CI = (D)1/(Dx)1 + (D)2/(Dx)2, where (Dx)1 and (Dx)2 indicate the individual dose of triptolite and celastrol required to inhibit a given level of cell growth, and (D)1and (D)2 are the doses of triptolite and celastrol necessary to produce the same effect in combination, respectively [36–38].

### Cell cycle analysis

Cells were harvested and washed twice with cold phosphate-buffered saline (PBS), then fixed with ice-cold 70% ethanol for 30 min at 4°C. After centrifugation at 200 × g for 10 min, cells were washed twice with PBS and resuspended with 0.5 ml PBS containing PI (50 μg/ml), 0.1% Triton X-100, 0.1% sodium citrate, and DNase-free RNase (100 μg/ml), and detected by FCM after 15 min incubation at room temperature in the dark. Fluorescence was measured at an excitation wavelength of 480 nm through a FL-2filter (585 nm). Data were analyzed using ModFit LT 3.0 software (Becton Dickinson) [39].

### Apoptosis assay

Cell apoptosis was evaluated with flow cytometry (FCM) assay. Briefly, cells were harvested and washed twice with PBS, stained with Annexin V-FITC and propidium iodide (PI) in the binding buffer, and detected by FACSCalibur FCM (BD, CA, USA) after 15 min incubation at room temperature in the dark. Fluorescence was measured at an excitation wave length of 480 nm through FL-1 (530 nm) and FL-2 filters (585 nm). The early apoptotic cells (Annexin V positive only) and late apoptotic cells (Annexin V and PI positive) were quantified [40, 41].

### Reactive oxygen species (ROS) assay

Cells were incubated with 10 μM of DHE for 30 min at 37°C, washed twice with PBS and immediately photographed under fluorescent microscope (Olympus, Japan) under the same conditions for each group. For each well, 5 fields were taken randomly and relative ROS levels were quantified by normalizing the net average intensity values of other groups to control group. [25, 42].

### Short interfering RNA (siRNA) assay

The sense sequences of HSP70, HSP27 and negative control siRNAs were: 5′-GAAGGACGAGUUUGAGCAC-3′, 5′-ACGGUCAAGACCAAGGAUG-3′, 5′-CCUACGC CACCAAUUUCGU-3′, respectively, and they were synthesized by Shanghai GenePharma. Each siRNA solution was mixed gently with the respective volume of the X-tremeGENE siRNA Transfection Reagent and allowed to form transfection mixture for 20 min. Cells were cultured in 6-well plate with DMEM until 50% of confluence, and added with the transfection mixture for 48 h before the next experiment [43, 44].

### Western blot analysis

Cells were harvested and washed twice with cold PBS, then resuspended and lysed in RIPA buffer (1% NP-40, 0.5% sodium deoxycholate, 0.1% SDS, 10 ng/ml PMSF, 0.03% aprotinin, 1 μM sodium orthovanadate) at 4°C for 30 min. Lysates were centrifuged for 10 min at 14,000 × g and supernatants were stored at −80°C as whole cell extracts. Total protein concentrations were determined with Bradford assay. Thirty μg proteins of each sample were separated on 12% SDS-PAGE gels and transferred to polyvinylidene difluoride membranes. Membranes were blocked with 5% BSA and incubated with the indicated primary antibodies. Corresponding horseradish peroxidase-conjugated secondary antibodies were used against each primary antibody. Proteins were detected using the chemiluminescent detection reagents and films [45, 46].

### Nude mice xenograft tumor assay

Balb/c nude mice were obtained from the Guangdong Medical Laboratory Animal Center and maintained with sterilized food and water. Six female nude mice with 5 weeks old and 20 g weight were used for each group. Each mouse was injected subcutaneously with H1299 or H157 cells (3 × 10^6^ in 100 μl of medium) under the shoulder. When the subcutaneous tumors were approximately 0.5 × 0.5 cm^2^ (two perpendicular diameters) in size, mice were randomized into four groups, and were injected intraperitoneally with vehicle alone (0.9% saline), triptolide alone (0.4 mg/kg), celastrol alone (2 mg/kg), or a combination of triptolide and celastrol every two day. The body weights of mice and the two perpendicular diameters (A and B) of tumors were recorded. The tumor volume (V) was caculated according to the formula:

$$\text{V}=\frac{\text{π}}{6}={\left(\frac{\text{A}+\text{B}}{2}\right)}^{3}$$

The mice were anaesthetized after experiment, and tumor tissue was excised from the mice and weight. The rate of inhibition (IR) was calculated according to the formula [47]:

$$\text{IR}=1-\frac{\text{Mean tumor weight of experimental group}}{\text{Mean tumor weight of control group}}\times 100\%$$

### Statistical analysis

A student's *t*-test was used to compare individual data points among each group. A *P*-value of <0.05 was set as the criterion for statistical significance.

## SUPPLEMENTARY FIGURES AND TABLE


