# Supplementary material for: Synergistic anticancer effects of triptolide and celastrol, two main compounds from thunder god vine
Source: Oncotarget. 2015 Oct 5;6(32):32790–804. doi: 10.18632/oncotarget.5411 (PMC4741730; doi:10.18632/oncotarget.5411)
Supplement: Supplementary file 1 [file oncotarget-06-32790-s001.pdf]

SUPPLEMENTARY FIGURES AND TABLE

A

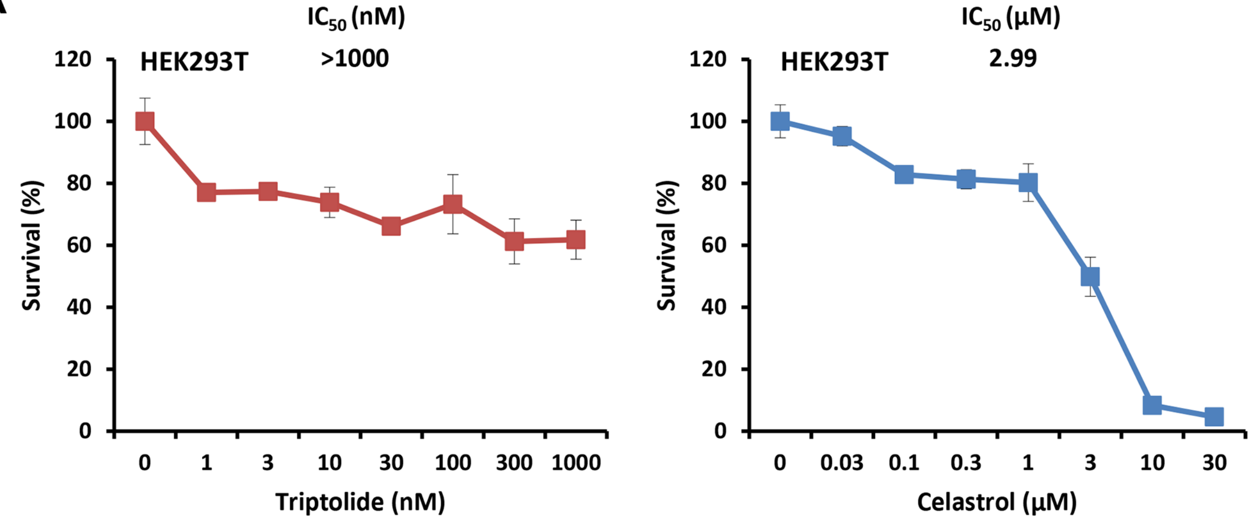

B

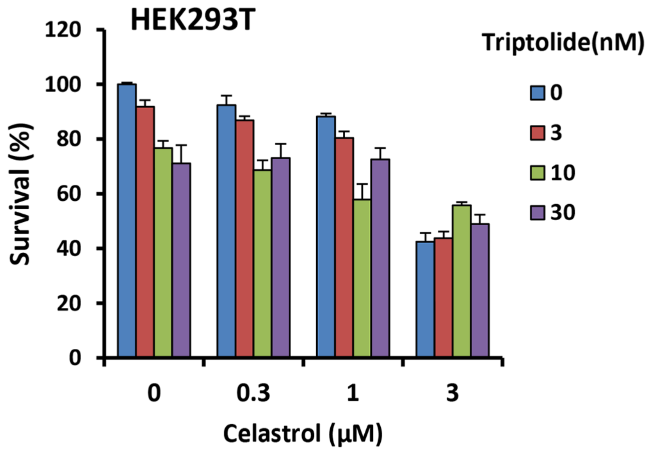

CI Data for Non-Constant Combo: T-C (Trip+Cela)

Dose Trip	Dose Cela	Effect	CI
3.0	0.3	0.86	1.00760
3.0	1.0	0.8	1.32816
3.0	3.0	0.43	0.79191
10.0	0.3	0.68	0.52058
10.0	1.0	0.57	0.57123
10.0	3.0	0.55	1.29812
30.0	0.3	0.73	1.71039
30.0	1.0	0.72	2.09237
30.0	3.0	0.49	1.22825

Supplementary Figure S1: Effects of alone or co-treatment with triptolide and celastrol on the growth of HEK293T cells *in vitro*. Human normal embryonic kidney cells HEK293T were treated with the indicated concentrations of triptolide and celastrolfor 72 h, and cell survival was detected by MTT assay. The summary survival curves **A.** growth histogram and CI values **B.** were shown.

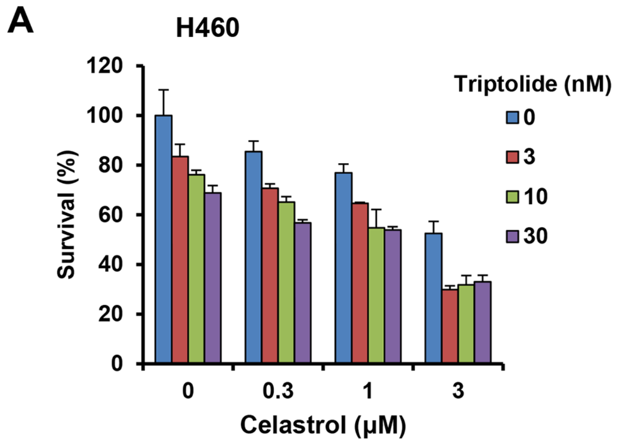

CI Data for Non-Constant Combo: T-C (Trip+Cela)

Dose Trip	Dose Cela	Effect	CI
3.0	0.3	0.7	0.35962
3.0	1.0	0.65	0.64197
3.0	3.0	0.3	0.21371
10.0	0.3	0.65	0.37517
10.0	1.0	0.57	0.43336
10.0	3.0	0.31	0.23094
30.0	0.3	0.57	0.33178
30.0	1.0	0.52	0.39261
30.0	3.0	0.33	0.27226

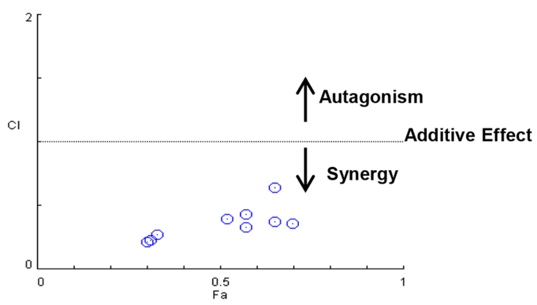

Combination Index Plot

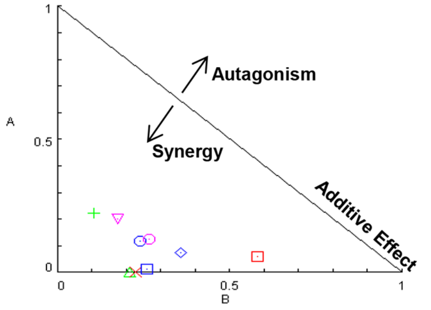

Normalized Isobologram for Combo

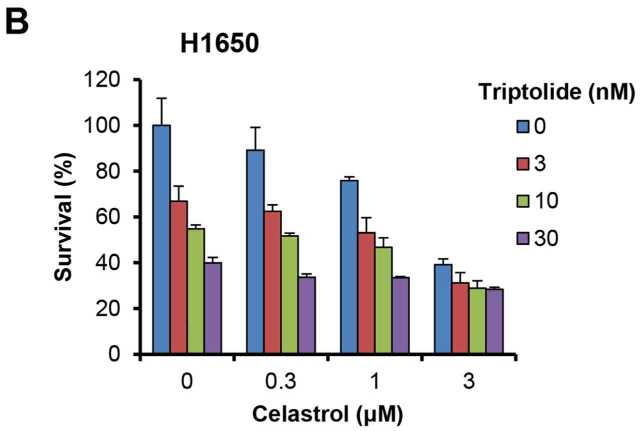

CI Data for Non-Constant Combo: T-C (Trip+Cela)

Dose Trip	Dose Cela	Effect	CI
3.0	0.3	0.62	0.81277
3.0	1.0	0.53	0.77567
3.0	3.0	0.31	0.68259
10.0	0.3	0.5	0.86446
10.0	1.0	0.43	0.75056
10.0	3.0	0.25	0.56325
30.0	0.3	0.33	0.57570
30.0	1.0	0.32	0.68322
30.0	3.0	0.27	0.81620

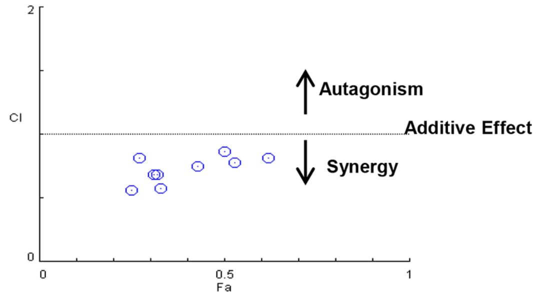

Combination Index Plot

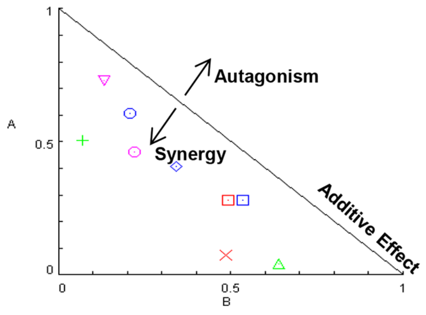

Normalized Isobologram for Combo

Supplementary Figure S2: Triptolide and celastrol synergistically inhibit the growth of other used cancer cells *in vitro.* H460 **A.** H1650 **B.** H1944 (*Continued*)

C

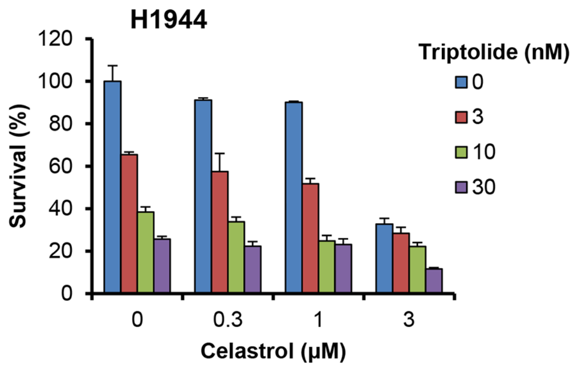

CI Data for Non-Constant Combo: T-C (Trip+Cela)

Dose Trip	Dose Cela	Effect	CI
3.0	0.3	0.57	0.84256
3.0	1.0	0.52	0.94176
3.0	3.0	0.28	0.69046
10.0	0.3	0.34	0.70263
10.0	1.0	0.25	0.51366
10.0	3.0	0.22	0.71413
30.0	0.3	0.22	0.87212
30.0	1.0	0.23	1.04902
30.0	3.0	0.12	0.55419

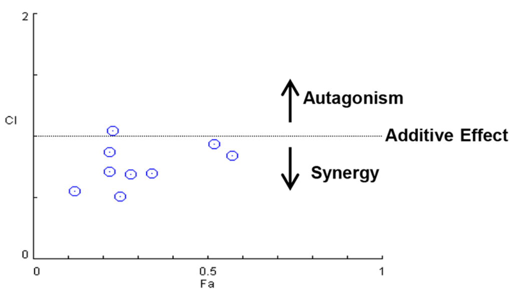

Combination Index Plot

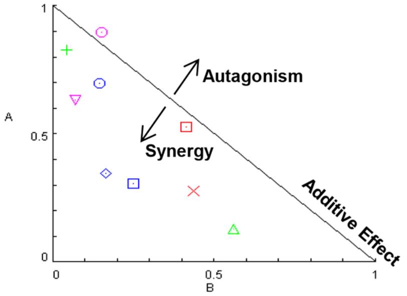

Normalized Isobologram for Combo

D

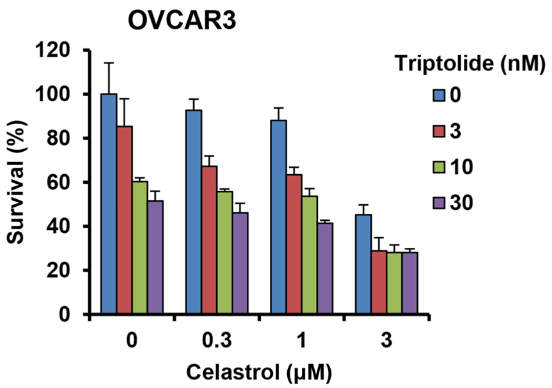

CI Data for Non-Constant Combo: T-C (Trip+Cela)

Dose Trip	Dose Cela	Effect	CI
3.0	0.3	0.67	0.46342
3.0	1.0	0.63	0.71124
3.0	3.0	0.29	0.46853
10.0	0.3	0.56	0.63284
10.0	1.0	0.54	0.81597
10.0	3.0	0.28	0.51986
30.0	0.3	0.46	0.97751
30.0	1.0	0.41	0.90368
30.0	3.0	0.28	0.72254

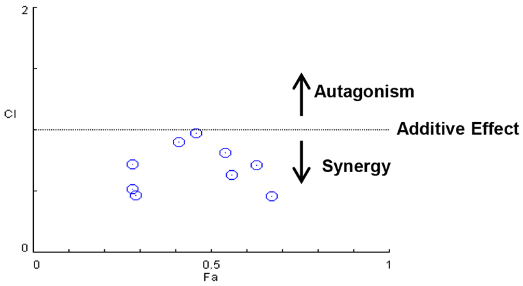

Combination Index Plot

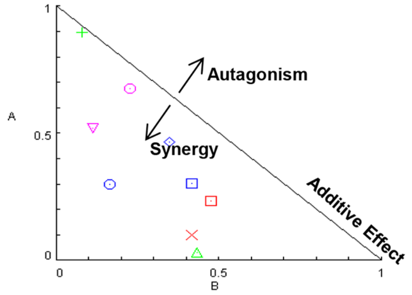

Normalized Isobologram for Combo

Supplementary Figure S2: (*Continued*) Triptolide and celastrol synergistically inhibit the growth of other used cancer cells *in vitro*. H460 **C.** OVCAR3 **D.** SKOV3 (*Continued*)

E

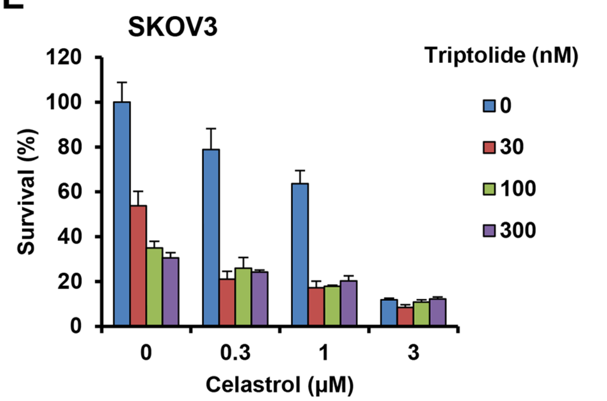

CI Data for Non-Constant Combo: T-C (Trip+Cela)

Dose Trip	Dose Cela	Effect	CI
30.0	0.3	0.21	0.16165
30.0	1.0	0.17	0.36799
30.0	3.0	0.08	0.57634
100.0	0.3	0.26	0.38636
100.0	1.0	0.18	0.44231
100.0	3.0	0.11	0.75356
300.0	0.3	0.24	0.68583
300.0	1.0	0.2	0.71313
300.0	3.0	0.12	0.86034

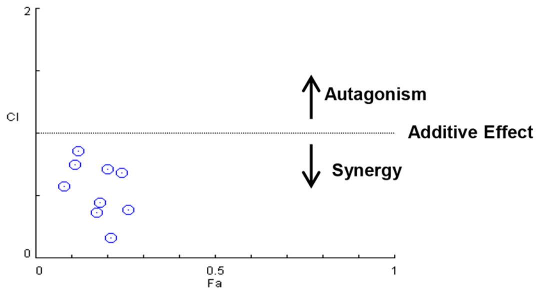

Combination Index Plot

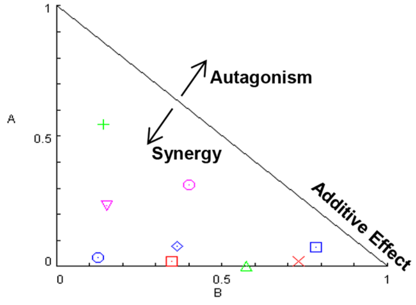

Normalized Isobologram for Combo

F

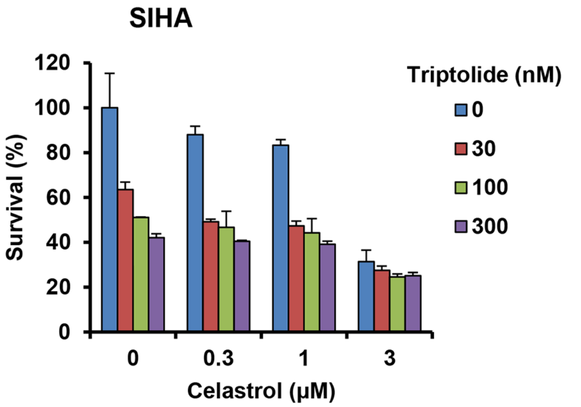

CI Data for Non-Constant Combo: T-C (Trip+Cela)

Dose Trip	Dose Cela	Effect	CI
30.0	0.3	0.49	0.32241
30.0	1.0	0.47	0.57771
30.0	3.0	0.27	0.63702
100.0	0.3	0.47	0.61289
100.0	1.0	0.44	0.72918
100.0	3.0	0.25	0.60093
300.0	0.3	0.4	0.72652
300.0	1.0	0.39	0.88108
300.0	3.0	0.25	0.65470

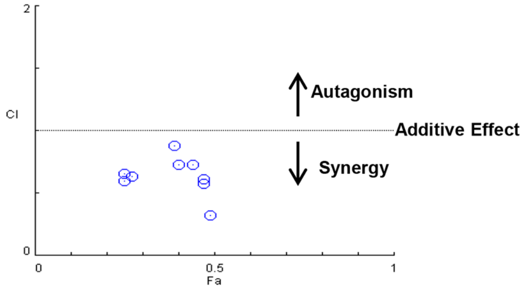

Combination Index Plot

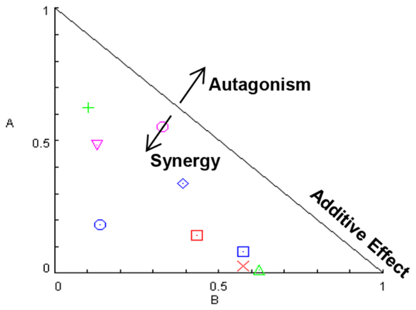

Normalized Isobologram for Combo

Supplementary Figure S2: (*Continued*) Triptolide and celastrol synergistically inhibit the growth of other used cancer cells *in vitro.* H460 **E.** SIHA **F.** HELA (*Continued*)

G

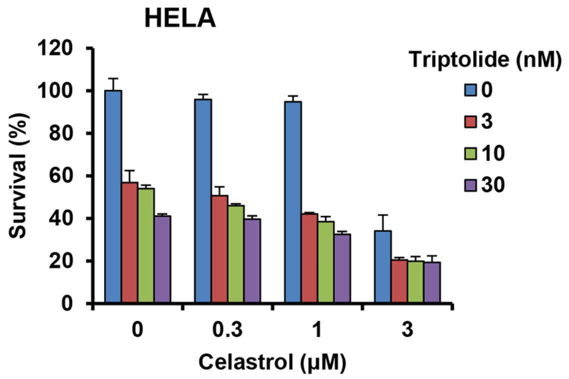

CI Data for Non-Constant Combo: T-C (Trip+Cela)

Dose Trip	Dose Cela	Effect	CI
3.0	0.3	0.5	0.38564
3.0	1.0	0.42	0.36997
3.0	3.0	0.2	0.44434
10.0	0.3	0.46	0.62243
10.0	1.0	0.38	0.41605
10.0	3.0	0.2	0.44882
30.0	0.3	0.39	0.64299
30.0	1.0	0.32	0.40387

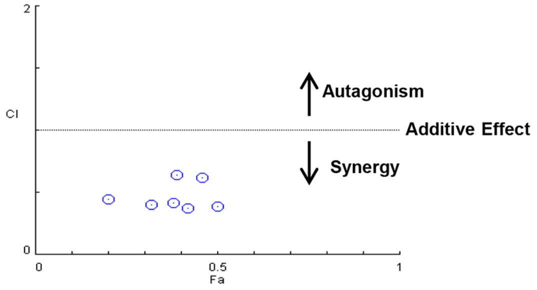

Combination Index Plot

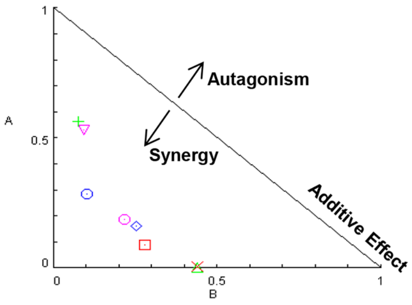

Normalized Isobologram for Combo

H

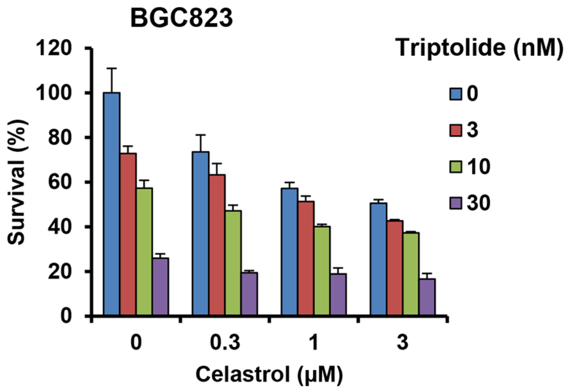

CI Data for Non-Constant Combo: T-C (Trip+Cela)

Dose Trip	Dose Cela	Effect	CI
3.0	0.3	0.63	0.89040
3.0	1.0	0.53	0.80925
3.0	3.0	0.42	0.72692
10.0	0.3	0.43	0.74762
10.0	1.0	0.37	0.62868
10.0	3.0	0.37	0.84797
30.0	0.3	0.19	0.55383
30.0	1.0	0.19	0.56336
30.0	3.0	0.17	0.50137

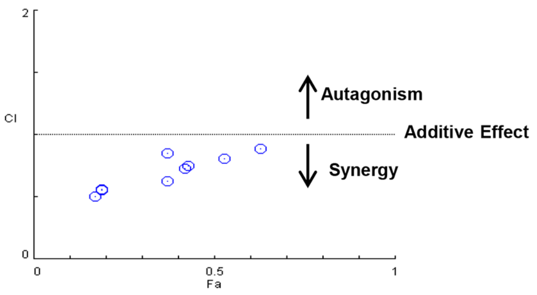

Combination Index Plot

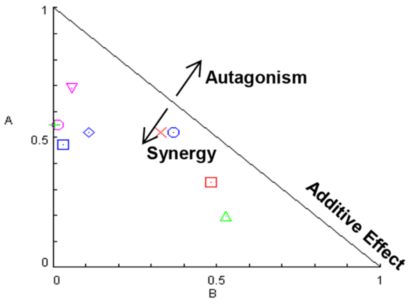

Normalized Isobologram for Combo

Supplementary Figure S2: (*Continued*) Triptolide and celastrol synergistically inhibit the growth of other used cancer cells *in vitro*. H460 **G.** BGC823 **H.** BEL7402 (*Continued*)

I

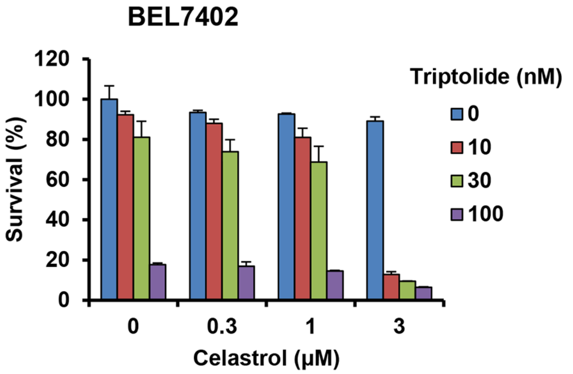

CI Data for Non-Constant Combo: T-C (Trip+Cela)

Dose Trip	Dose Cela	Effect	CI
10.0	0.3	0.88	0.58512
10.0	1.0	0.8	0.41577
10.0	3.0	0.13	0.06923
30.0	0.3	0.73	1.00713
30.0	1.0	0.68	0.88319
30.0	3.0	0.09	0.16584
100.0	0.3	0.17	0.82217
100.0	1.0	0.15	0.75803
100.0	3.0	0.06	0.43539

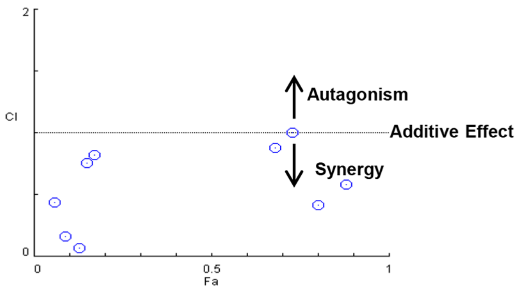

Combination Index Plot

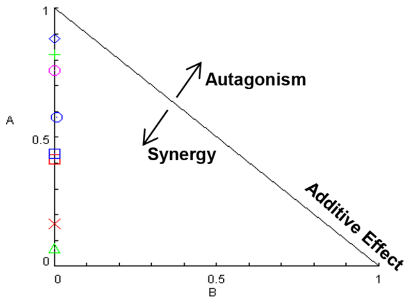

Normalized Isobologram for Combo

J

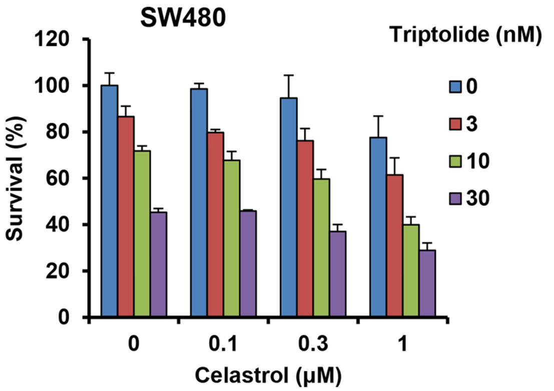

CI Data for Non-Constant Combo: T-C (Trip+Cela)

Dose Trip	Dose Cela	Effect	CI
3.0	0.1	0.8	0.64286
3.0	0.3	0.76	0.66812
3.0	1.0	0.61	0.64228
10.0	0.1	0.68	0.95888
10.0	0.3	0.59	0.71209
10.0	1.0	0.39	0.44645
30.0	0.1	0.46	1.01630
30.0	0.3	0.37	0.71535
30.0	1.0	0.29	0.58072

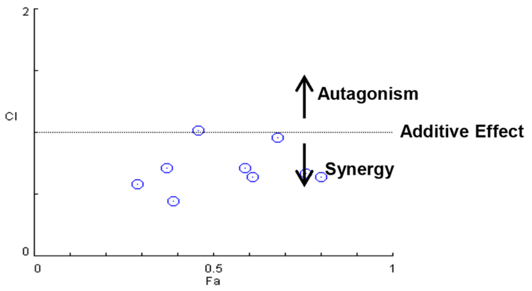

Combination Index Plot

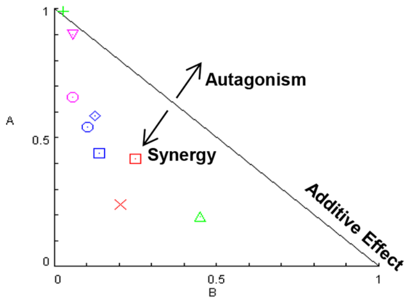

Normalized Isobologram for Combo

Supplementary Figure S2: (*Continued*) Triptolide and celastrol synergistically inhibit the growth of other used cancer cells *in vitro*. H460 **H.** BEL7402 **I.** and SW480 **J.** cells were treated with the indicated concentrations of triptolide and celastrol.for 72 h, and cell survival was detected by MTT assay. The summary growth histogram, dose-effect curve, CI values and normalized isobologram of three independent experiments were shown.

Supplementary Table S1: The quantified data of Protein expressions

Figure 3C	H1299				H157			
	Control	Trip	Cela	Trip + Cela	Control	Trip	Cela	Trip + Cela
CDK1	1.00	1.22	1.23	1.54	1.00	1.03	0.84	1.02
CDK2	1.00	0.89	0.79	0.49	1.00	0.48	0.50	0.24
CDK4	1.00	0.76	0.94	0.50	1.00	0.73	0.84	0.43
CDK6	1.00	0.77	0.95	0.33	1.00	0.46	0.70	0.50
Cyclin B	1.00	1.29	1.17	1.39	1.00	1.55	0.82	0.99
Cyclin D	1.00	0.61	0.69	0.18	1.00	0.70	0.38	0.16
Cyclin E	1.00	0.44	0.66	0.21	1.00	0.89	0.74	0.52
p21	1.00	2.01	2.34	2.87	1.00	1.14	1.09	1.31
p27	1.00	0.95	1.09	0.62	1.00	0.40	0.37	0.08
Rb	1.00	0.71	1.08	0.83	1.00	0.59	0.40	0.33
pRb	1.00	0.59	0.82	0.37	1.00	0.81	0.77	0.50

Figure 4D	H1299				H157			
	Control	Trip	Cela	Trip + Cela	Control	Trip	Cela	Trip + Cela
PARP	1.00	0.71	1.15	0.70	1.00	0.95	0.70	0.33
C-PARP	1.00	1.85	1.71	3.09	1.00	1.17	0.56	1.20
Caspase-3	1.00	0.84	1.04	0.78	1.00	0.96	0.61	0.38
C-Caspase-3	1.00	1.08	0.75	1.76	1.00	1.52	0.99	1.48
Bcl-2	1.00	0.69	1.02	0.29	1.00	1.42	0.89	0.42
Bcl-XS/L	1.00	0.93	0.90	0.70	1.00	1.09	0.69	0.57
Bax	1.00	0.91	0.97	0.44	1.00	0.57	0.42	0.26
Mcl-1	1.00	0.32	0.65	0.15	1.00	0.53	1.29	0.60
XIAP	1.00	0.38	1.24	0.03	1.00	0.34	0.80	0.35
Survivin	1.00	0.44	0.48	0.29	1.00	0.77	0.80	0.56

Figure 6C	H1299				H157			
	Control	Trip	Cela	Trip + Cela	Control	Trip	Cela	Trip + Cela
HSP90	1.00	0.40	0.73	0.21	1.00	0.44	1.09	0.30
HSP70	1.00	0.80	1.25	0.45	1.00	0.46	1.55	0.30
HSP27	1.00	0.71	1.21	0.48	1.00	0.50	2.00	0.51
AHA1	1.00	0.44	0.66	0.27	1.00	0.75	1.06	0.32
AKT	1.00	0.67	0.61	0.41	1.00	0.74	0.99	0.41
CDC37	1.00	0.49	0.62	0.15	1.00	1.08	0.98	0.38
EGFR	1.00	0.57	0.85	0.29	1.00	0.54	0.85	0.37
E-Cadherin	1.00	0.73	0.67	0.51	1.00	1.09	1.39	0.51
MDM2	1.00	0.49	0.79	0.31	1.00	0.63	0.96	0.40

(*Continued*)

Figure 6C	H1299				H157			
	**Control**	**Trip**	**Cela**	**Trip + Cela**	**Control**	**Trip**	**Cela**	**Trip + Cela**
p65	1.00	0.66	0.93	0.25	1.00	0.31	0.80	0.26
RAF1	1.00	0.75	0.86	0.42	1.00	0.70	0.92	0.37
β-Catenin	1.00	0.54	0.69	0.53	1.00	0.92	1.36	0.43

The quantified data of protein expressions. The Western blot results of Figure 3C, 4D and 6C were quantified by Image J software. The protein levels in other groups were normalized to control group which was defined as 1.00.
